# Robust patterns in the stochastic organization of filopodia

**DOI:** 10.1186/1471-2121-11-86

**Published:** 2010-11-17

**Authors:** Asma N Husainy, Anne A Morrow, Theodore J Perkins, Jonathan M Lee

**Affiliations:** 1 Department of Biochemistry, Microbiology & Immunology, University of Ottawa, 451 Smyth Road, Ottawa, Ontario, K1H 8M5, Canada; 2 Ottawa Hospital Research Institute, 501 Smyth Road, Ottawa, Ontario, K1H 8L6, Canada

## Abstract

**Background:**

Filopodia are actin-based cellular projections that have a critical role in initiating and sustaining directional migration in vertebrate cells. Filopodia are highly dynamic structures that show a rich diversity in appearance and behavior. While there are several mathematical models of filopodia initiation and growth, testing the capacity of these theoretical models in predicting empirical behavior has been hampered by a surprising shortage of quantitative data related to filopodia. Neither is it clear how quantitatively robust the cellular filopodial network is and how perturbations alter it.

**Results:**

We have measured the length and interfilopodial separation distances of several thousand filopodia in the rodent cell line Rat2 and measured these parameters in response to genetic, chemical and physical perturbation. Our work shows that length and separation distance have a lognormal pattern distribution over their entire detection range (0.4 μm to 50 μm).

**Conclusions:**

We find that the lognormal distribution of length and separation is robust and highly resistant to perturbation. We also find that length and separation are independent variables. Most importantly, our empirical data is not entirely in agreement with predictions made based on existing theoretical models and that filopodial size and separation are an order of magnitude larger than what existing models suggest.

## Background

When mammalian cells migrate, they do so by generating protrusive actin structures in the form of advancing lammellipodia or filopodia [1,2]. The lamellipodium is a broad cellular extension composed of a mesh-like network of crosslinked actin fibers. Filopodia, on the other hand, are finger-like cellular projections composed of a core of actin filaments bundled in a parallel array [3,4]. Filopodia are the first cellular structures to reach new space during cell migration and their growth factor receptors guide movement towards chemoattractants [5]. Filopodial adhesion molecules also provide traction [6]. During migration, filopodia are often overtaken by advancing lamellipodia and filopodial actin bundles contribute to the formation of contractile structures within the cell body [7]. Filopodia have an important role in controlling cell migration *in vivo *and are essential for neurogenesis in mice and for cell-cell adhesion during Drosophila embryogenesis [3,4]. Filopodia are also involved in cancer progression, as many filopodial proteins are known to regulate tumor invasion and metastatic development [8,9].

The simple composition of filopodia belies the complex biochemical events that shape their initiation and growth. The pathways controlling the assembly of mature filopodia are controversial, and two different models, convergent elongation and *de novo *nucleation, compete for general acceptance [3,4,10]. During convergent elongation, linear actin bundles in the lamellipod, termed microspikes, fuse into a lambda-shaped structure that becomes a filopodium as is grows outward from the plasma membrane [10,11]. In *de novo *nucleation, filopodia are created by actin nucleating proteins at or near the plasma membrane and are independent of lamellar actin [4,10,12]. Experimental evidence supports both models and it therefore seems likely that there are multiple mechanisms of filopodia initiation.

In mammalian cells, filopodia have a strikingly varied appearance and behavior. Their lengths span greater than two orders of magnitude and they can grow to 50 μm or more in size [3,4]. Filopodial behavior is also highly variable, and filopodia in the same cell are observed undergoing phases of growth, retraction or stasis. The velocity of growth and retraction is variable, and filopodia can have velocities ranging from 0.25-1 μm/minute [13]. Several theoretical models have been used to describe filopodia formation and growth [14-20]. Parameters that have been incorporated into these models include the number of actin filaments in a filopodium, plasma membrane elasticity, G-actin concentration, actin retrograde flow, actin depolymerization and the mechanical strength of the actin polymers [14-20]. These studies make predictions as to the length distribution of filopodia and interfilopodial separation distances. However, there is a surprising paucity of quantitative data related to these parameters. In addition, it is unclear how perturbation quantitatively affects the filopodial system. In this report, we have measured the length and distance separation of several thousand filopodia in the non-transformed rodent cell line Rat2. Analysis of this data indicates that filopodia length and interfilopodial distance are distributed lognormally and this distribution is highly robust and resistant to perturbation.

## Methods

### Cell lines and treatments

Rat2 fibroblasts were purchased from American Type Culture Collection (Manassa, VA) and cultured in Dubecco's Modified Eagle Medium High Glucose 1X from Gibco, Invitrogen (Grand Island, NY) containing 10% Fetal Bovine Serum (FBS) (Gibco) and 1% antibiotic/antimycotic (Gibco). The cultured cells were incubated in 10 cm plates at 37°C in 5% CO→_2_. Cells were treated with bradykinin at 100 ng/ml for 30 minutes using DMSO as a vehicle. For poly-D-lysine experiments, cover slips were coated with 50 μg/ml poly-D-lysine for 2 hours prior to cell plating. Rat2 fibroblast cells ectopically expressing PI4KIIIβ and empty vector controls have been previously described [21,22].

### Immunofluorescence

Rat2 cells were grown to 70-80% confluency, trypsinized with 0.05% 1X Trypsin-EDTA (Gibco), diluted 1:100 and plated in 6 well plates containing glass coverslips (Fisher; Pittsburg, PA). 24 hrs later, cells were fixed in 3.7% paraformaldehyde for 20 minutes, permeabilized with 0.5% Triton-X for 15 minutes and left overnight in IF Buffer (130 mM NaCl, 7 mM Na→_2_HPO_4_, 3.5 mM NaH_s_PO_4, _7 mM NaN_3_, 0.2% Triton X-100, 0.1% BSA, 0.05% Tween-20, ph 7.4). The following day, cells were stained for 1 hr with Phalloidin-488 (Invitrogen) diluted 1:200 in 1X PBS (pH7.4) and subsequently stained with Hoescht-405 (Invitrogen) diluted 1:40 in 1X PBS. Coverslips were mounted on glass microscope slides (Fisher) with Fluorescent Mounting Medium (Dako; Carpinteria, CA). Images of single Rat2 cells were obtained from an Olympus Fluoview FV1000 laser scanning confocal microscope. Openlab Software (Improvision, MA) was used to measure filopodia lengths and separation.

### Data Analysis

For each length or distance data set, histograms were plotted on a logarithmic axis, with bins of equal width in log-space. For both visualization and statistical fitting purposes, as described below, the empirical cumulative distribution function, F(x), is defined as the fraction of the data having a value strictly less than x. The empirical probability density function, which was used only for visualization purposes, was taken to be the Parzen windows estimator with a radius parameter of h = 0.25 applied in the log-transformed space. That is, if x_1 _... x_n _are the original data and y_1 _... y_n _are the transformed data (y_i _= log_10_x_i_) then the probability density function is f(y) = c(y)/n, where c(y) is the number of points y_1 _... y_n _for which the absolute difference to point y is less than or equal to h.

We fit different distributions to the data by comparison of idealized and empirical cumulative distribution functions. Let G(x, θ) denote the cumulative distribution function of a statistical distribution with parameter or parameters θ. We judged that filopodia lengths or interfilopodial distances less than 0.4 μm could not be reliably quantified from the images. So, no such measurements were included in our data set. To fit θ based on the data we first defined the "cut-off cumulative distribution function" as G_C_(x, θ) = 0 if x ≤ 0.4 and G_C_(x, θ) = (G(x, θ)-G(0.4, θ))/(1-G(0.4, θ)) if x > 0.4. The cut-off function recognizes that our data collection procedure does not record any values smaller than 0.4 μm; in essence, any part of the statistical distribution falling below that threshold is zeroed out and the remainder of the distribution is rescaled so that it integrates to one. We define the error of parameters θ as the sum of squared residuals: E(θ) = Σ_X_(F(x)- G_C_(x, θ)), where the sum is over x = 10^-0.40^, 10^-0.39^, 10^-0.38^, ..., 10^1.60 ^for length data and x = 10^-0.40^, 10^-0.39^, 10^-0.38^, ..., 10^2.30 ^for distance data. Parameters θ are fit by minimizing the error E(θ). We fit four different families of distributions in this way: the exponential, which has probability density function g(x, λ) = λ exp(-λx) and cumulative distribution function G(x, λ) = 1- exp(-λx); the powerlaw, which has probability density function g(x, x_min_, α) = ((α-1)/x_min_)(x/x_min_)^-α ^and cumulative distribution function G(x, x_min_, α) = 1 - (x/x_min_)^1-α ^for x ≥ x_min_; the Gaussian, which has probability density function g(x, μ, σ) = (2πσ^2^)^-1/2 ^exp(-(x-μ)^2^/2σ^2^); and the lognormal, which has probability density function g(x, μ, σ) = (2πσ^2^x^2^)^-1/2 ^exp(-(ln(x)-μ)^2^/2σ^2^) for x > 0.

## Results

### Quantitation of Filopodia

**Figure 1:**
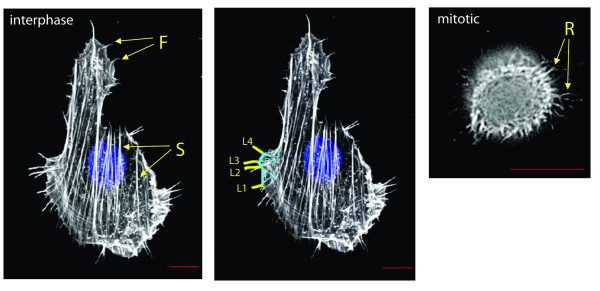
**The actin cytoskeleton in Rat2 cells**. Rat2 fibroblast cell stained for actin (white) and DNA (blue). The leftmost panel shows transverse actin stress fibers (S) and filopodia (F) as hair-like projections from the cell perimeter. The central panel shows counting of individual filopodia lengths (L1, L2, L3, L4) and distance separation (D1, D2, D3). The right panel shows a mitotic cell with retraction fibers (R) indicated. The red scale bar is 10 μm.

Filopodia span a wide range of observable lengths and individual cells show high variability in the size and number of filopodia they possess. To understand filopodia in their cellular context, we observed filopodia production in rodent fibroblast Rat2 cells. We chose this cell line because it is non-cancerous and individual cells have filopodia that span nearly two orders of magnitude in length. The appearance of the actin cytoskeleton in typical Rat2 cells is shown in Figure 1. In interphase, two types of linear actin polymers are commonly seen, stress fibers (S) and filopodia (F). Stress fibers traverse the cell in a lengthwise manner. Filopodia, on the other hand, are visible as linear projections from the cell body that emanate from multiple places and proceed in multiple directions. Filopodia are distinguishable from the less frequently observed and visibly similar retraction fibers. Retraction fibers are seen primarily in mitotic cells but also appear in cells in interpahse, at the trailing edge during migration. Based on our previously published work with living Rat2 cells [23], filopodia can be visually distinguished from retraction fibers (R) based on their relative thickness and extended presence behind the plasma membrane. We have purposely excluded mitotic cells from our analysis to avoid potential confusion between filopodia and retraction fibers. Moreover, Rat2 cells are relatively non-migratory so they have very few retraction fibers relative to filopodia in non-mitotic cells.

To quantitate filopodial properties in Rat2 cells, we used image analysis software to manually trace the lengths of individual filopodia in fixed Rat2 cells. The length of a filopodium was extrapolated from the pixel length of the trace line. Based on the resolution of our fluorescence microscopy system, we estimate that we can accurately determine the length of filopodia > 0.4 μm in length. Filopodia shorter than this cannot accurately be distinguished from lamellar actin structures and therefore were not counted. We also measured the distance that separates a given filopodium from its nearest neighbor. Cells visualized were non-mitotic and not visibly attached to other cells but were otherwise randomly chosen. The cell population as a whole was in a logarithmic phase of growth and no attempt was made to synchronize filopodia growth cycles. As such, the filopodia that we measure represent structures in undetermined phases of growth, shrinkage and stasis. We collected this data for all filopodia in the individual cells that we imaged. Thus, each filopodium is defined by a length (L*_x_*) and a separation distance (D_x_) measurement.

### Filopodia lengths are distributed lognormally

**Figure 2:**
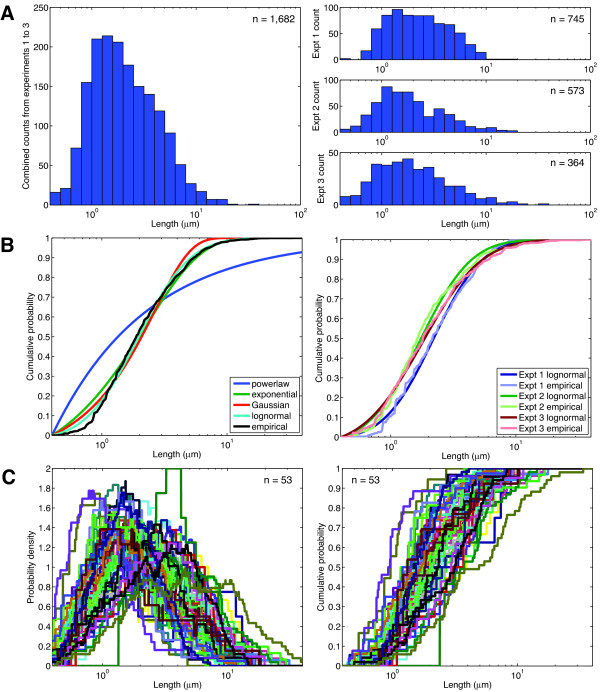
**Filopodia length distribution is unimodal and best fits a lognormal model**. **(A) **Histogram of all the filopodia lengths (n = 1,682), and independent experiments 1, 2 and 3 with n = 745, n = 573 and n = 364 respectively. **(B) **Empirical cumulative distribution functions (CDFs) of lengths with the lognormal and other statistical models for all the experiments combined and each independent experiment 1, 2 and 3. **(C) **Empirical PDFs and CDFs of filopodia lengths for 53 cells.

We compiled filopodia length measurements from three independent experiments. We counted filopodia from a total of 52 Rat2 cells (experiment 1 = 25; experiment 2 = 18; experiment 3 = 10). The total number of filopodia was 1,682 (experiment 1 = 745; experiment 2 = 573; experiment 3 = 364). As shown in Figure 2A, filopodia distribution in the total data set is unimodal with a mean of 2.70 μm. The length distribution of the individual experiments was also unimodal with a respective mean of 2.79 μm, 2.49 μm, and 2.84 μm for Experiments 1, 2 and 3. Approximately 82% of the filopodia fall within the range of 1 μm to 10 μm in length.

We next determined the statistical model that would best fit the empirical cumulative probability distribution (CDF) of filopodia lengths and distances. We found that the length distribution of the collective dataset was best modeled as a lognormal distribution (p(x)= (2πσ^2^x^2^)^-1/2 ^exp(-(ln(x)-μ)^2^/2σ^2^)) (Figure 2B). That is, the logarithm of the length is approximately normally distributed. The dataset is poorly modeled as an exponential (p(x) = λ exp(-λx)), Gaussian (p(x) = (2πσ^2^)^-1/2^exp(-(x-μ)^2^/2σ^2^)) or power law (p(x) = ((α-1)/x_min_)(x/x_min_)^-α^) distribution (Figure 2B). The power law and exponential distributions fit least well, as they are incapable of capturing the unimodality of the observed data. The exponential, however does provide a reasonable fit for the distribution of filopodia larger than ~1.5 μm. The Gaussian is the next most accurate, capturing the unimodal data, but it overestimates the left tail while underestimating the right tail. The lognormal captures both unimodality and the heavy right tail. The datasets of individual experiments are also fit well by lognormal distributions (Figure 2A, B), as are the length distributions from each individual cell (Figure 2C). The similarity in CDF distribution between individual cells in a population indicates that the system regulating filopodia length shows robust behavior in the Rat2 population.

### Filopodia distance separations are distributed lognormally

**Figure 3:**
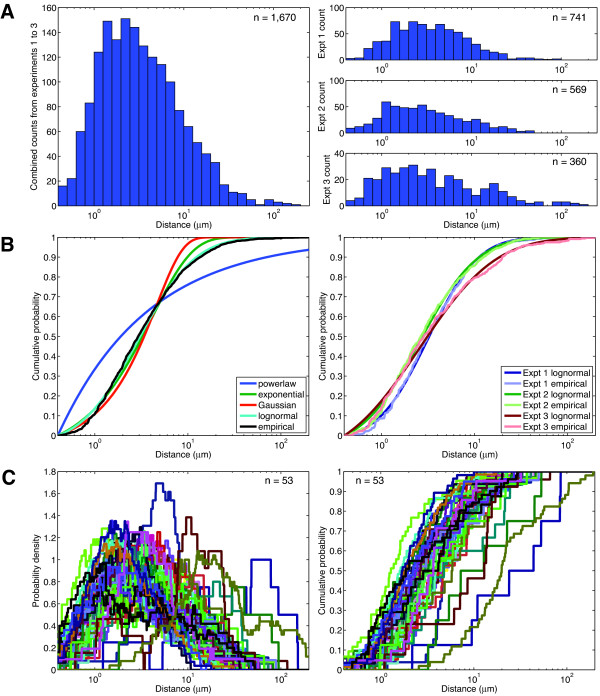
**Filopodia distance separation distribution is unimodal and best fits a lognormal model**. **(A) **Histogram of all the filopodia distances (n = 1,670), and independent experiments 1, 2 and 3 with n = 741, n = 569 and n = 360 respectively. **(B) **Empirical cumulative distribution functions (CDFs) of distances with the lognormal and other statistical models for all the experiments combined and each independent experiment 1, 2 and 3. **(C) **Empirical PDFs and CDFs of filopodia distances for 53 cells.

As we did for filopodia lengths, we compiled the data for the separation distances between adjacent filopodia. As with filopodia length, the separation distance is unimodal in both the total data set and in the three separate experiments (Figure 3A). The mean distance for the collective dataset was 6.18 μm and experiments 1, 2 and 3 had respective means of 5.52 μm, 5.11 μm, and 9.23 μm. When we calculated the CDF for the distance distribution, lognormal was the best fit of the distribution data (Figure 3B). As is the case of filopodial length, the separation dataset is poorly modeled as an exponential, Gaussian, or power law distribution (Figure 3B). The power law distribution is the poorest fit, while an exponential distribution may fit the distribution of filopodia that are separated by 10 μm or more. Nearly all of the cells in a Rat2 population show a good lognormal fit of separation distance data. The similarity in CDF distribution between individual cells in a population indicates that the system regulating filopodia distance separation shows robust behavior between cells. 74% of the interfilopodial distance separation falls within the range of 1 μm to 10 μm.

### Length and separation distance are independent variables

**Figure 4:**
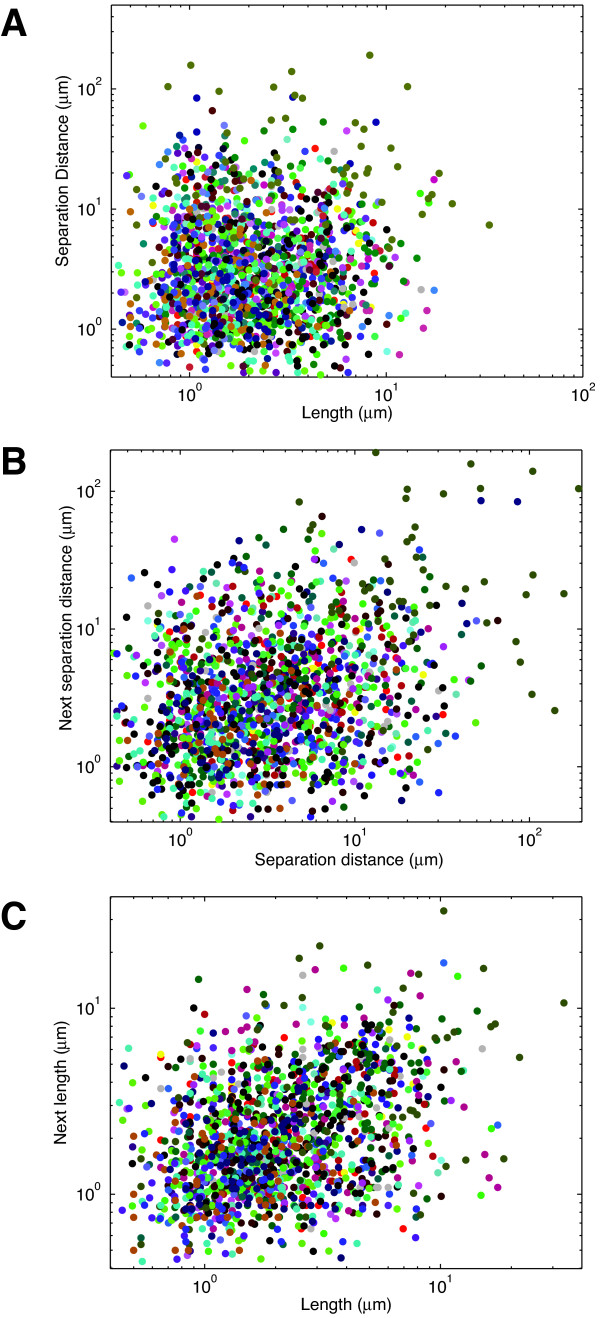
**Filopodia length and distance separation are independent**. **(A) **Scatter plot of filopodia lengths against the neighboring distances shows no correlation. Identically coloured data points represent measurements from the same cell. **(B) **Scatter plot of the distances between filopodia versus the distances between the next filopodia in a clockwise direction around the edge of the cell. **(C) **Scatter plot of the lengths of filopodia versus the lengths of the next filopodia in a clockwise direction around the edge of the cell.

The polymerization of actin polymers within a filopodium depends on an intracellular pool of G-actin. It is possible that as an individual filopodium grows, it might locally deplete the G-actin pool around it and thereby interfere with *de novo *filopodia creation or actin polymerization in pre-existing filopodia. If this were the case, then there may be some empirical relationship between filopodia length and separation. To test this idea, we determined whether or not the length of an individual filopodium is detectably correlated with separation distance between its neighbours (Figure 4A). The figure shows the length of individual filopodia versus the average separation distance between its two nearest filopodia plotted on a log-log scale. On the whole, however, there is no substantial correlation between filopodial length and interfilopodial separation distance (r~0.02). On a per cell basis, there are some cells that show a weak negative correlation between length and separation (r~-0.6) and some with a weak positive correlation (r~0.25). We next determined whether or not there was any substantial correlation between the distance separating adjacent filopodia and whether there might be correlation between the lengths of filopodial neighbours. Such a correlation would be predicted should the concentration of a G-actin pool be a limiting factor in either the initiation of an individual filopodium or in its total length. Figure 4B shows that there is a mild correlation between the separation distance of adjacent filopodia. A similar weak correlation exists between the length of filopodial neighbours (Figure 4C). This suggests that any spatial constraints linking filopodia length and separation are likely to be quite small and, together with Figure 4A, suggests that filopodial length and separation distance are likely to be independent variables.

### Perturbation Analysis of Filopodia

We next wanted to investigate how the filopodia system quantitatively responds to perturbation. There are many agents that have been described to be inducers of filopodia formation, but high-quality empirical measurement of what these agents do to filopodia are not common. Since we have been able to mathematically describe the filopodia system with some degree of confidence, we are now able to define how known filopodial perturbations affect the system as a whole. We chose to alter filopodia production in three distinct manners: genetically, chemically and physically. For the genetic perturbation, we engineered Rat2 cells to ectopically express the lipid kinase PI4KIIIβ, which we have reported stimulates filopodia production [22]. To chemically induce filopodia, we used the peptide hormone bradykinin, which induces filopodia through activation of G-protein coupled receptors [24]. To physically induce filopodia, we coated the growth substrate with poly-D-lysine, which could increase filopodia size by increasing the positive charge of the substrate and enhancing adhesion.

**Figure 5:**
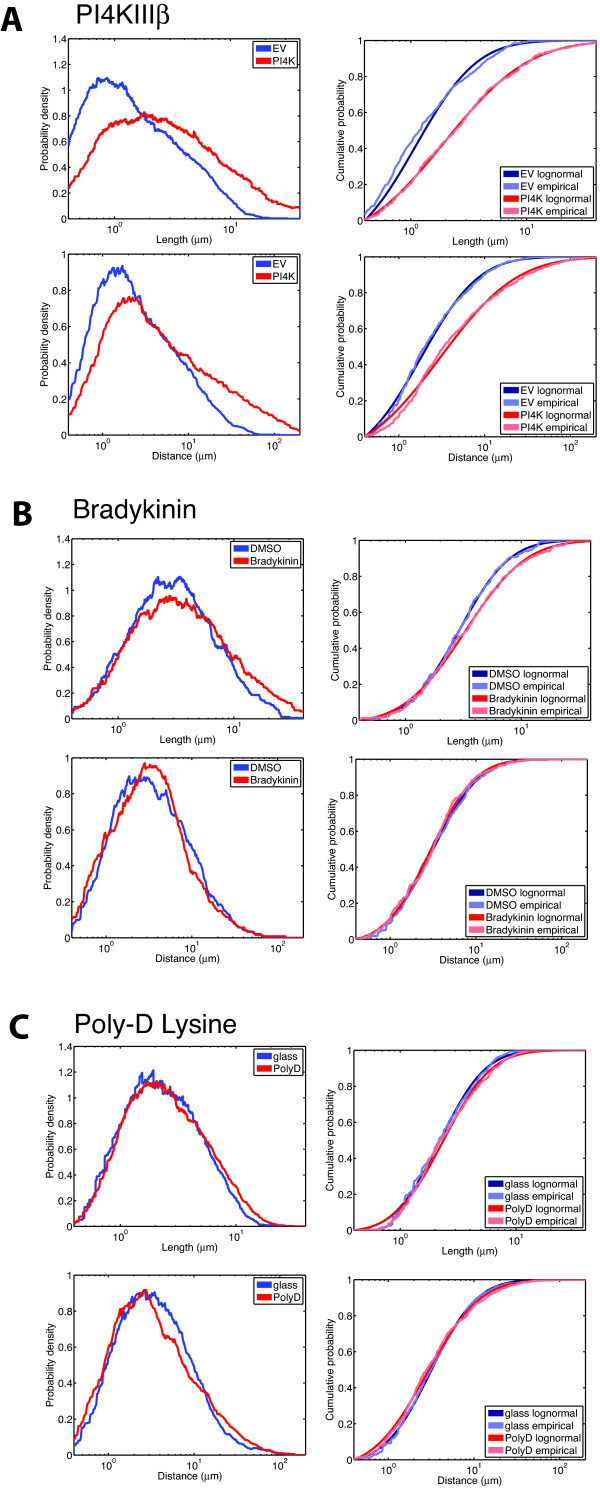
**Robust nature of the filopodial length and distance separation lognormal distribution**. Perturbation analysis of Rat2 cells after genetic, chemical and physical induction of filopodia. **(A) **PПI4KIIIβ expression induces filopodia and increases both length and separation distance relative to the empty vector control. Rat2 cells stably expressing PI4KIIIβ and controls have been previously described [22]. **(B) **Bradykinin, a chemical inducer of filopodia, increased the length but had no effect on the interfilopodial distances compared to the DMSO control. Rat2 cells were treated with 100 ng/mL bradykinin. **(C) **Poly-D-lysine, a physical inducer of filopodia, increased the length of filopodia modestly but had no effect on distances.

As shown in Figure 5A, expression of PI4KIIIβ causes a large increase in the length of filopodia. The mean length in PI4KIIIβ-expressing cells was 5.13 μm, significantly longer than the 2.03 μm mean length in the vector-only controls (t-test, p < 0.0001). The length distribution remains unimodal, and an increase in the number of long filopodia (10 μm - 100 μm) is visible. The longest filopodium in PI4KIIIβ expressing cells was 65.71 μm. Interestingly, the separation distance between the filopodia also increases following PI4KIIIβ expression and the mean separation in PI4KIIIβ-expressing cells was 12.00 μm, significantly higher than the 4.16 μm distance in vector-only controls (t-test, p < 0.00005). Importantly, even though the length and separation of filopodia have increased substantially, the distribution of both parameters remains lognormal. This indicates that the lognormal distribution is a robust aspect of filopodia length and separation distance control.

Bradykinin treatment causes an increase in filopodia length, albeit to a much lesser extent than PI4KIIIβ expression. The mean length of bradykinin treated filopodia was 5.19 μm, significantly longer than the 3.95 μm mean length in DMSO treated controls (t-test, p < 0.04773). The change that bradykinin makes to filopodia length distribution is primarily in the longer filopodia as 27% of filopodia in bradykinin treated cells were > 6 μm in length, compared to only 17% (48/282) of filopodia in the DMSO controls. The mean separation did not change appreciably, with a mean distance of 4.97 μm in the bradykinin-treated cells compared to 5.16 μm in the DMSO-treated cells. The lack of significant change in distance separation (t-test, p < 0.4386) further strengthens our assertion that filopodia length and distance separation are independent variables. As in the case with PI4KIIIβ expression, the distributions of length and separation following bradykinin remain unimodal and are best fit by lognormal distributions.

The effects of poly-D-lysine on filopodia were very modest (Figure 5C). The mean length of filopodia in cells grown on poly-D-lysine was 3.13 μm which is not much longer than the 2.82 μm mean filopodia length of Rat2 cells grown on plain glass slides. This difference is statistically significant (t-test, p < 0.02968) due to the large number of samples, but it is not readily apparent in the PDF and CDF distribution. Like bradykinin, no change in filopodia separation distances is apparent (5.90 μm compared to 5.49 μm). The distribution of filopodia is unimodal and fits a lognormal distribution. Collectively, the effects of genetic, chemical and physical filopodia inducers show that increases in filopodia length do not apparently alter the lognormal distribution pattern of filopodia length nor the lognormal distribution of the distances that separate them.

**Figure 6:**
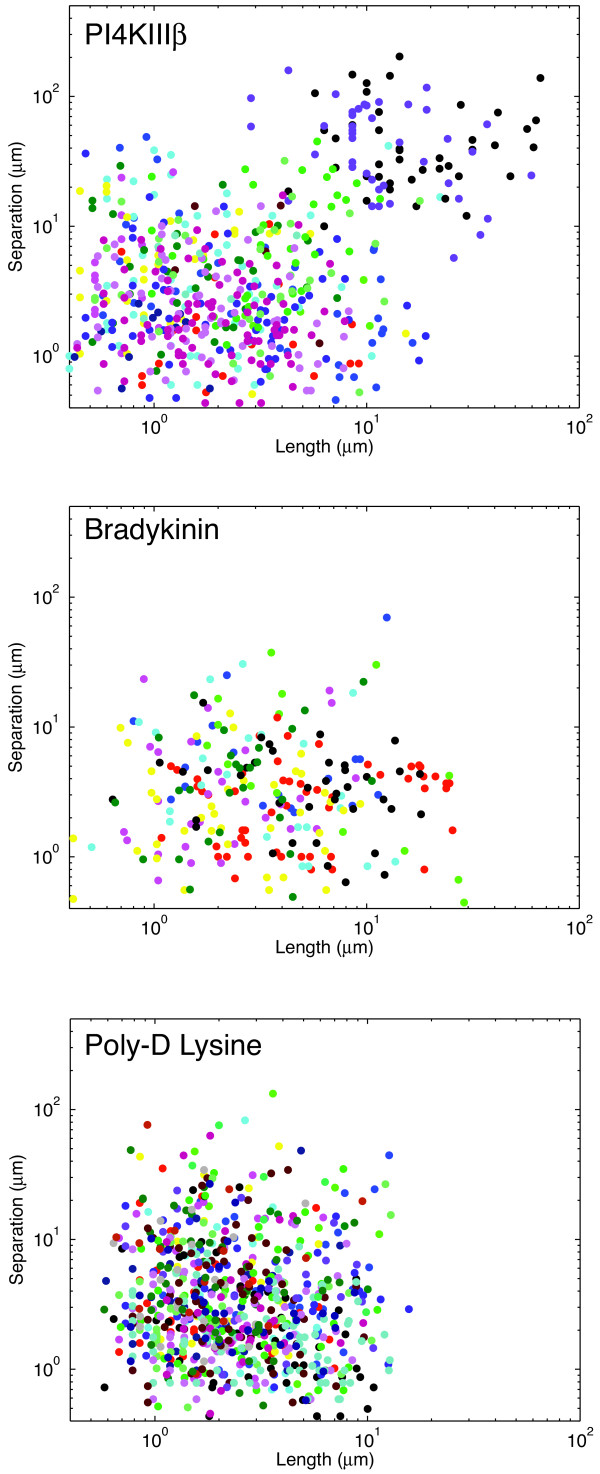
**Filopodia length and distance separation are independent after perturbation**. Graph of filopodial length and separation distance for PI4KIIIβ, Bradykinin and Poly-D-lysine perturbations. Identically coloured data points represent measurements from the same cell.

Lastly, we chose to analyze the relationship between length and separation distance in the perturbed cells. Figure 6 shows this relationship, plotted on a log-log scale, for all three perturbations. In the case of bradykinin and poly-D lysine, there was no obvious relationship between length and separation. In this respect, these two perturbations do not cause changes from the wild-type situation. In the case of PI4KIIIβ expression, there is a weak, albeit statistically significant, positive correlation (r~0.39). This appears to result from two individual cells (coloured black and purple) with very long and highly separated filopodia. Filopodia length and separation are not highly correlated in these two cells, but the magnitude of the length and separation measurements leads to an apparent correlation in the overall population. As such, we conclude that length and filopodial separation remain independent variables even following perturbation.

## Discussion and Conclusions

Among the goals for this study was to identify a method to quantitatively describe the filopodial system in a given cell population. Our interest in this idea first arose when we began to quantitate the effect that PI4KIIIβ expression had on filopodia in the mammalian breast cancer cell line BT549 [22]. PI4KIIIβ is one of four mammalian kinases, PI4KIIα, PI4KIIβ, PI4KIIIα and PI4KIIIβ, which generate PI4P (Phosphatidylinositol 4-phosphate) from PI (phosphatidylinositol) [25,26]. Our work with this kinase and an oncogenic protein that activates it, eEF1A2, suggested that eEF1A2 and PI4KIIIβ stimulated filopodia production by activating the production of PI(4,5)P_2 _[21-23]. PI(4,5)P_2 _abundance regulates filopodia by recruiting actin-remodeling proteins to the migratory leading edge [27]. While the effect that PI4KIIIβ expression had on filopodia was visually striking and qualitatively apparent, quantitative description proved difficult because of the highly variable appearance of filopodia in a given cell population. Filopodia numbers vary per cell; their lengths in a single cell frequently span more than an order of magnitude and very long filopodia sometimes appear even in unstimulated cells. In the end, we adopted a system that approximated our qualitative visual evaluation [22]. We scored cells that had 10 or more filopodia > 3 μm in length as positive and the remainder as negative [22]. Based on this criterion, PI4KIIIβ expression has a demonstrable and significant numerical effect on filopodia production [22]. However, this descriptive system was unsatisfying because there is nothing intrinsically unique about filopodia longer than 3 μm nor is having 10 or more long filopodia of obvious biologic importance. In this study, we hoped to identify objective and quantitative parameters of the filopodial system to determine whether or not any given stimulus was altering filopodia production. Based on our current analysis, we propose that μ the peak of the density of the lognormal distribution represents a useful quantitation parameter of the filopodial network. The robust nature of the lognormal distribution (Figure 2) among independent replicates of the same cell population indicates that it appears to be a tightly regulated feature of the cell type. Moreover, individual cells of the same population have a similar CDF distribution (Figure 2C). Based on our analysis of the bradykinin, poly-D-lysine perturbation, and PI4KIIIβ expression, we believe that counting ~300 filopodia in a population will allow quantitative determination of the effect that a given stimulus has on filopodial appearance based on alterations in μ.

The extensive filopodia length data that we have collected provide an empirical base on which to test existing theoretical models of filopodia growth [14-16]. Our empirical data does not closely match many existing theoretical models. For example, Lan and Papoian predict that the frequency distribution of filopodia lengths will be tight and will peak at ~0.6 μm [16] while our empirical peak is ~3 μm. Moreover, we frequently observe filopodia >5 μm in length, which is not readily accounted for in their work. The presence of these long filopodia is also not in agreement with Atligan et al., who propose that mechanical buckling forces provide strong limits on filopodia growth beyond a length of 1.7 μm [14]. It is possible that adhesion between filopodia and the growth substrate may reduce the effect that buckling forces have in retarding filopodial growth, but this remains to be empirically tested. While Mogilner & Rubinstein predict that most of the filopodia will be of 2 μm in length [15], in closer agreement with our studies, their modeling does not account for the lognormal distribution of the filopodial nor the large numbers of long filopodia that we observe. Mogilner & Rubinstein postulate that three different parameters limit filopodia growth dependent on filopodial length [15]. According to their model, membrane resistance limits filopodia below 0.4 μm in length, between 0.4-1.5 μm filopodia length is limited by buckling, while longer filopodia growth is limited by the diffusion of G-actin. More recent modeling has suggested that the generation of long filopodia (~4-6 μm length) may be the result of active G-actin transport within filopodia or the loss of capping protein function [17,18]. However, we consistently observe filopodia in the > 6 μm range, therefore we hypothesize that additional factors must be at work.

It is worth mentioning that existing models of filopodia formation are based on the assumption that the actin filaments within an individual filopodium are as long as the filopodium itself [14-16]. Because of the directionality of the filopodial actin fibers, filopodial growth directly reflects actin polymerization at the tips. Many studies support this model [4,10,12], but a recent cryo-electron tomographic analysis of filopodia in *Dictyostelium *suggests otherwise [28]. This ultrastructural analysis indicates that filopodia are composed of discontinuous actin filament bundles ~100 nm in length. The discontinuous nature of the actin filaments within these filopodia could therefore allow for longer filopodia because the buckling forces that affect individual filaments would be predicted to be smaller. However, the commonality of this structure in filopdia in other cell types remains to be determined.

It is important to note some limitations in our current study. Firstly, we have relied exclusively on the Rat2 cell line and other cells may behave differently. The B16 melanoma line is commonly used to study filopodia and these cells show much smaller filopodia and more uniform length distribution relative to Rat2 [13]. The cell-type specificity of filopodial quantitative parameters indicates that differing biochemical pathways are at play in individual cell lines. Another limitation of our study is that we have not measured filopodia in living cells. Individual filopodia undergo phases of growth, stasis and retraction during their lifespan [13]. Our use of fixed (non-living) cells, treats filopodia as stationary objects and, in a sense, ignores their dynamism. To help circumvent this issue, we have collected data from a large population of cells. Because we have made no attempt to synchronize or otherwise manipulate the filopodial growth cycle, our collective dataset represents filopodia in all their dynamic phases. Large-scale analysis of filopodia in living cells will be necessary to betterunderstand and measure filopodial dynamics.

The inter-filopodial distance separation data that we collected also allow sus to test the predictions made by Mogilner et al. [15]. Mogilner based their model on previous work by Svitkina et al. [11], which provided evidence that filopodia are initiated from the fusion of cytosolic actin fibers. These lamellar actin fibers fuse into a λ-shaped precursor and subsequent actin polymerization creates a filopodium. Based on the distribution of λ-precursors and their lateral motion, Mogilner modeled interfilopodial spacing to a range of 1-3 μm, with a tight distribution. Another theoretical study, based on the idea that membrane protein adhesion complexes regulate the initiation of protrusive structures, also suggests that filopodia will have separation distances in this range [29]. However, we observe that filopodia are often widely spaced, frequently having separation distances of 10 μm or more. It should be noted that lambda precursors are not the only proposed pathway of filopodia initiation, and filopodia may also form from *de novo *nucleation by Formin proteins independent of lamellar actin strands [4,10,12]. Moreover, filopodial fusion, an event predicted [14,20] but not yet reported may also function to increase inter-filopodial distances. These may account for our large filopodial spacing.

We were initially surprised to find that PI4KIIIβ expression not only increases filopodial length, but also increases their separation (Figure 5). Since no biochemical regulators of interfilopodial separation have been identified to date, it is not immediately apparent how PI4KIIIβ increases this parameter. However, it is possible that concomitant with an increase in filopodial length, PI4KIIIβ may be depleting a pool of G-actin or actin polymerizing factors that control filopodial initiation. On the other hand, our work indicates that filopodia length and separation are independent variables (Figure 4), suggesting they are regulated by different mechanisms. This is also further buttressed by our observation that PI4KIIIβ affects both length and separation, while bradykinin and poly-D-lysine only affect filopodial length.

We find that both filopodial length and separation distance have a lognormal distribution. Earlier biophysical modeling of filopodia-like structures in lymphocytes shorter than 1.1 μm has suggested the restraining force of the membrane might account for a heavy right-tailed length distribution [20]. In this work, a Gaussian distribution accounts for filopodia up to ~0.3 μm in length and then an exponential distribution of longer filopodia generates a heavy right tail. Qualitatively, this is consistent with our data. The presence of some experimental skew even in our log-transformed length dataset indicates that the lognormal does not wholly account for the data. Indeed, fitting a distribution of the type described by Gov [27] results in a tighter fit to the data (results not shown). However, it is not clear that the biophysical model studied by Gov has relevance to our data, as the lengths that we observe are an order of magnitude larger than the ones modeled by Gov. Moreover, we found that much of the skew in the log-transformed data is due to cell-to-cell variation. Some individual cells showed positive skew, but others showed negative skew. As such, we did not feel there was sufficient support to adopt the four-parameter Gov model [27] or even a three-parameter skew-lognormal model for filopodial length distribution.

The lognormal distribution is not uncommon in biology. For example, species abundance distributions and long-term survival in breast cancer are lognormal functions [30,31]. Fruit and flower sizes also show lognormal distributions [32]. With respect to filopodia, the cellular significance of lognormal length and separation distance distributions is unclear. However, this distribution is highly robust and resistant to perturbation. PI4KIIIβ expression and bradykinin treatment affect filopodia length but do not change the lognormal distribution. This suggests that the lognormal distribution is robust and likely reflects strong biophysical constraints on the pathways controlling filopodial dynamics. Filopodia length distribution is lognormal from the smallest length that we confidently detect (0.4 μm) up to almost 100 μm. Generally speaking, a lognormal distribution can arise as the product of a number of random variables. There are dozens, perhaps hundreds of proteins that affect actin polymerization and higher-ordered polymer assembly [1,2]. The biochemical mechanism(s) through which their combinatorial action creates a lognormal distributed function is unclear to us. Nevertheless, fluctuations in the concentrations of these proteins may be among the factors influencing filopodia length and separation. Membrane forces are also likely to be important [19,20]. Further theoretical modeling of the actin cytoskeleton is likely to be necessary to resolve this issue.

## Authors' contributions

ANH carried out the cell biology studies, performed the microscopy and measurements, participated in the data analysis and drafted the manuscript; AAM generated cell lines, assisted in the cell biology, participated in the microscopy and measurements and assisted in manuscript revision; TJP participated in the design of the study, performed the mathematical analysis of the data, participated in manuscript writing and revision and generated the figures; JML conceived of the study, participated in its design and coordination, participated in the data analysis and participated in manuscript writing and figure generation. All authors read and approved the final manuscript.

## Acknowledgements

Supported by grants from the Natural Sciences and Engineering Research Council of Canada (JML, TJP). AAM is supported by a fellowship from the Canadian Institute of Health Research. We thank D. Bickel, J. Copeland, H. McBride, S. Michnick, D. Pinke, and S. Shaikh for helpful discussion and reading of this manuscript. We also thank H. McBride for help with and use of the confocal microscope.
